# Odontogenic Infection Complicated by Cervicofacial Necrotizing Fasciitis in a Healthy Young Female

**DOI:** 10.7759/cureus.16835

**Published:** 2021-08-02

**Authors:** Amanda Cecchini, Cody J Cox, Arthur A Cecchini, Krupa Solanki, Roger McSharry

**Affiliations:** 1 Internal Medicine Residency, East Tennessee State University Quillen College of Medicine, Johnson City, USA; 2 Internal Medicine, East Tennessee State University Quillen College of Medicine, Johnson City, USA; 3 Pulmonology and Critical Care, East Tennessee State University Quillen College of Medicine, Johnson City, USA

**Keywords:** cervicofacial, necrotizing, fasciitis, head and neck, infection, odontogenic

## Abstract

Necrotizing fasciitis (NF) is a critical and rapidly progressive infection of the skin and soft tissue, and it is associated with a high mortality rate. NF of the cervicofacial region is uncommon due to the rich vascular supply of the head and neck, which promotes an efficient immune response to infection. Patients who are immunocompromised or have comorbidities affecting the vasculature, such as diabetes mellitus or peripheral vascular disease, are at an increased risk of more severe disease and outcome. Cervicofacial necrotizing fasciitis (CNF) is most frequently attributed to mucosal damage, such as those related to dental infections or local trauma including medical procedures. Due to its ability to quickly spread to the neck and mediastinum, CNF must be diagnosed and treated expeditiously. In this report, we present a case of a 28-year-old female with a past medical history significant for obesity and tobacco abuse who presented to the emergency department (ED) with fever, left-sided facial pain, cervical pain, and swelling. She had worsening symptoms despite current treatment with clindamycin for a dental abscess. A CT scan of the head and neck revealed an odontogenic abscess complicated by CNF. Intravenous antibiotics were initiated and she underwent prompt surgical intervention. She remained nasally intubated following her surgery due to concern for postoperative edema leading to airway compromise. Following extubation, she experienced an uncomplicated recovery. This case demonstrates that NF is a complication of dental infection that may occur even in young and relatively healthy patients. Additionally, due to the swiftly destructive nature and high mortality rate of CNF, early diagnosis and aggressive medical and surgical therapy are essential to reduce morbidity and mortality.

## Introduction

Necrotizing fasciitis (NF) is a serious infection that leads to rapid necrosis of subcutaneous tissue. NF of the head and neck is rare but carries a high mortality rate. Dental infection appears to be the most common site of origin; however, involvement of the neck, mediastinum, and chest wall commonly occurs due to the infection’s rapid ability to spread. Patients with comorbidities commonly associated with NF, such as diabetes, advanced age, peripheral vascular disease, and renal failure, usually have increased mortality and overall poorer outcomes. In this report, we present a young patient without any significant risk factors who developed NF of the head and neck from a wisdom tooth abscess. Thanks to early diagnosis, the patient promptly received appropriate medical therapy and surgical intervention, leading to a successful outcome.

## Case presentation

A 28-year-old female with an unremarkable past medical history presented to the ED with a chief complaint of left-sided facial pain and swelling. She had consulted her dentist five days prior to the presentation for left-upper wisdom tooth pain and had been diagnosed with a dental abscess, for which she had been prescribed 150 mg clindamycin three times daily for seven days. Despite complying with her antibiotic regimen, the patient experienced worsening face and neck swelling, pain, headache, and vomiting, with a fever as high as 102.7 °F.

**Figure 1:**
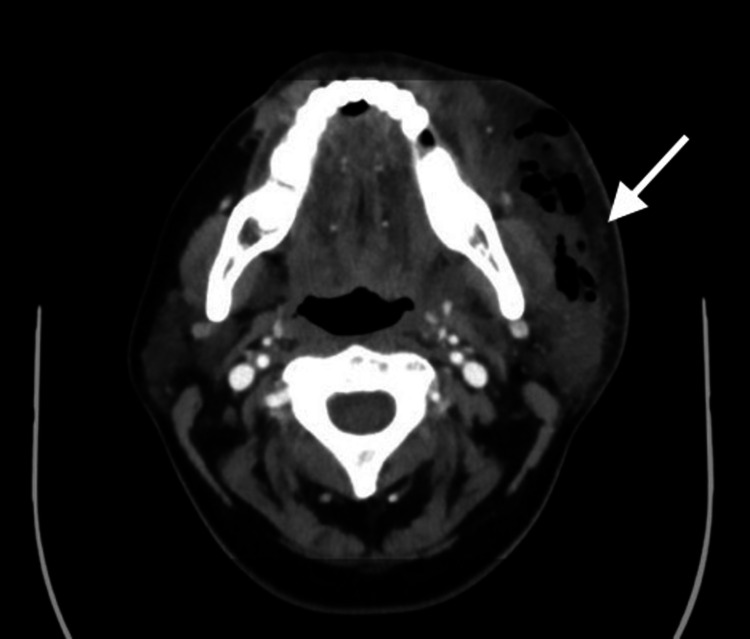
CT scan of the patient's sinuses, facial bones, and neck The scan revealed subcutaneous stranding in numerous pockets of air throughout the left face and neck without fluid collection (arrow) CT: computed tomography

On arrival at the ED, she was afebrile with a temperature of 97.6 °F, a heart rate of 112 beats per minute, and a blood pressure of 133/77 mmHg. Her oxygen saturation was 100% on room air. Her labs were significant for leukocytosis, anemia, and hypokalemia with a white blood cell count of 28.5 K/uL, hemoglobin of 11.8 g/dL, and potassium of 2.7 mmol/L. Her lactate was normal at 1.4 mmol/L. She underwent a contrast-enhanced CT scan of the sinuses, facial bones, and neck, which revealed subcutaneous stranding in numerous pockets of air throughout the left face and neck without fluid collection, which seemed to be originating from the left-upper wisdom tooth (Figure 1). 

She was given 900 mg intravenous (IV) clindamycin, IV vancomycin, and 10 mg IV dexamethasone as well as pain and nausea medication. Oral surgery and otolaryngology were consulted. The following morning, she underwent extraction of her left-upper wisdom tooth with incision, drainage, and debridement of local submandibular, buccal, and masseteric space abscesses. To allow optimal surgical access, the patient was intubated nasally for the procedure. Following surgical debridement, she remained intubated due to concern for postoperative edema compromising the airway. She was extubated on postoperative day two without complication.

Intraoperative cultures grew Streptococcus anginosus, group F streptococcus, Eikenella corrodens, Prevotella, and Bacteroides species. Infectious disease was consulted, and they recommended a two-week course of linezolid and clindamycin. The patient was discharged on postoperative day four and found to have experienced significant improvement at her two-week clinical follow-up.

## Discussion

NF is a subcategory of necrotizing soft tissue infections (NSTIs), which also includes necrotizing cellulitis and necrotizing myositis. NSTIs demonstrate rapid onset, suppurative tissue destruction of skeletal muscle and the overlying fascia, epidermis, dermis, and subcutaneous fat requiring prompt diagnosis and treatment. Poor blood supply to fascia and subcutaneous fat allows these anatomical layers to be incredibly susceptible to NF. Albeit rare, the New England Journal of Medicine reports an annual incidence that ranges from 0.3-15.5 cases per 100,000 population depending on the region [1].

However, NF of the head and neck, termed cervicofacial necrotizing fasciitis (CNF), is exceptionally rare (5.3%) due to abundant vasculature that allows prompt immune response to infectious insult [2]. When CNF does occur, individuals may require up to 30 days of inpatient treatment due to the severity of the disease, with mortality rates as high as 44% if the infection spreads to the mediastinum [3,4]. CNF occurs most commonly with odontogenic infections; however, it is also associated with dental surgeries, major penetrating trauma, or any breach of the oropharyngeal mucosal barrier. Patients who are otherwise immunocompetent with no past medical history are typically unaffected. According to a systematic review of literature, prominent risk factors include chronic corticosteroid use, neutropenia, cirrhosis, alcoholism, obesity, and peripheral vascular disease [5]. Diabetes mellitus is associated with a particularly significant risk, as patients frequently have associated arteriosclerosis and immune compromise, preventing adequate blood flow to the area, resulting in an impaired immune response.

Ludwig’s angina is a similar soft tissue infection of the head/neck that is characterized by a rapidly spreading, bilateral cellulitis of the submandibular space without lymphatic spread. Like CNF, the most common cause is odontogenic infections; however, Ludwig’s angina is also associated with suppurative parotitis and peritonsillar abscess [6]. Cases have been reported in which CNF was incorrectly diagnosed as Ludwig’s angina due to the absence of palpable crepitus on physical exam, resulting in delayed surgical debridement until skin necrosis was observed [7]. Due to the rapid progression of NF, delay in diagnosis and initiation of treatment can lead to severe outcomes; therefore, practitioners must be vigilant in considering this differential.

**Figure 2:**
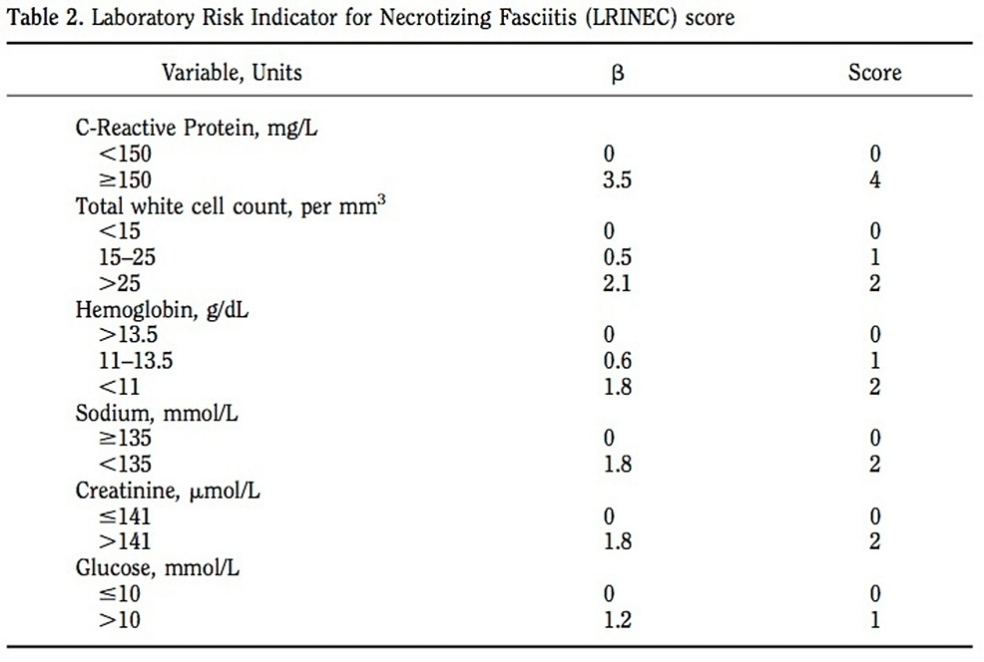
Laboratory Risk Indicator for Necrotizing Fasciitis

Once CNF is considered as a potential differential, practitioners should promptly review the Laboratory Risk Indicator for Necrotizing Fasciitis (LRINEC) score (Figure 2). LRINEC is a clinical tool that aids in differentiating NF from other soft tissue infections. This chart generates a score based on C-reactive protein (CRP) levels, white blood cell count, hemoglobin, sodium, creatinine, and glucose levels. A score of 6 or greater should raise the suspicion for NF, and a score of 8 or greater is strongly predictive [5]. In our patient, the CRP was not measured and therefore the LRINEC score was not calculated; however, even without knowing the CRP, she did have a score of at least 4 as she had leukocytosis, anemia, and hyperglycemia.

NF infections are further classified into polymicrobial (type I) or monomicrobial (type II) categories. Type I infections, caused by both anaerobic and aerobic bacteria, commonly affect older individuals with predisposing comorbidities and are responsible for the majority of CNF infections. Several types and combinations of anaerobic bacteria such as anaerobic streptococci, Bacteroides, and Fusobacterium have been isolated from soft tissue cultures. Type II infections account for a minority of CNF cases and are usually attributed to group A Streptococcus (GAS), other beta-hemolytic streptococci, and Staphylococcus aureus [methicillin-sensitive (MSSA) or methicillin-resistant (MRSA)] [4]. Type II infections are known to affect individuals of any age, regardless of immunocompetency. One study involving 5,400 cases of GAS infection revealed a case-fatality rate of almost 14%, with nearly one-quarter of these deaths associated with NF [8].

Clinical manifestations of CNF progress rapidly. A prompt diagnosis and a comprehensive treatment plan consisting of hemodynamic support, broad-spectrum antibiotics, and surgical debridement are critical to reducing mortality rates. Patients who present with signs of soft tissue infection, pain out of proportion to physical exam findings, and associated fever with or without hemodynamic instability should undergo emergent surgical exploration. In fact, withholding surgical debridement for antimicrobial monotherapy is associated with a survival rate of nearly 0% [9]. Physical exam may reveal thin, discolored skin accompanied by a necrotic, malodorous wound with or without discharge. Crepitus is an ominous indicator. The presence of gas located within soft tissues on CT is highly specific for necrotizing infections. However, surgical intervention should be performed emergently and not delayed for radiologic imaging. Blood cultures should be collected immediately, followed by prompt administration of empiric antibiotics. Surgical exploration must be performed to evaluate the extent of soft tissue destruction and to collect material for culture, gram stain, and pathologic analysis. All confirmed necrotizing infections require surgical debridement. Empiric antibiotic treatment for NF infections should consist of a broad-spectrum therapy that effectively covers both gram-negative and gram-positive bacteria, as well as anaerobes. Treatment should incorporate a three-part regimen: (1) a broad-spectrum agent such as a carbapenem or piperacillin-tazobactam, (2) an agent with MRSA coverage such as vancomycin, and (3) clindamycin, as it is suitable for protection against toxin-producing strains of bacteria such as streptococci and staphylococci [10]. Antibiotic therapy may then be de-escalated once the identification and sensitivities of the infectious organism emerge.

Hyperbaric oxygen (HBO) therapy has been evaluated in a number of studies as adjunctive therapy for necrotizing infections. One study demonstrated increased survival rates [11], as the treatment helps to delineate necrotic tissue to facilitate a safer and more precise debridement process [12]. Our patient showed improvement following antibiotics therapy and surgical intervention; therefore, HBO was not considered. However, it is a therapy to consider for severe cases of NF as it is associated with increased delivery of oxygen to ischemic tissues, which allows for more expeditious wound healing [11].

## Conclusions

NF is a critical complication of odontogenic infection, a diagnosis commonly encountered by medical practitioners. Our case demonstrates that NF may occur even in young and otherwise healthy populations. Prompt diagnosis is essential as the infection spreads rapidly and is often fatal. In cases of cervicofacial infection that does not respond to antibiotic treatment, NF should be ruled out as quickly as possible with a thorough physical exam and appropriate imaging such as a CT scan to avoid delay in treatment and reduce morbidity and mortality.

## References

[1] Stevens DL, Bryant AE (2017). Necrotizing soft-tissue infections. N Engl J Med.

[2] Carter LM, Layton S (2009). Cervicofacial infection of dental origin presenting to maxillofacial surgery units in the United Kingdom: a national audit. Br Dent J.

[3] Gunaratne DA, Tseros EA, Hasan Z (2018). Cervical necrotizing fasciitis: systematic review and analysis of 1235 reported cases from the literature. Head Neck.

[4] Mathieu D, Neviere R, Teillon C, Chagnon JL, Lebleu N, Wattel F (1995). Cervical necrotizing fasciitis: clinical manifestations and management. Clin Infect Dis.

[5] Wong CH, Khin LW, Heng KS, Tan KC, Low CO (2004). The LRINEC (Laboratory Risk Indicator for Necrotizing Fasciitis) score: a tool for distinguishing necrotizing fasciitis from other soft tissue infections. Crit Care Med.

[6] Boscolo-Rizzo P, Da Mosto MC (2009). Submandibular space infection: a potentially lethal infection. Int J Infect Dis.

[7] Becker M, Zbären P, Hermans R (1997). Necrotizing fasciitis of the head and neck: role of CT in diagnosis and management. Radiology.

[8] O'Loughlin RE, Roberson A, Cieslak PR (2007). The epidemiology of invasive group A streptococcal infection and potential vaccine implications: United States, 2000-2004. Clin Infect Dis.

[9] Anaya DA, Dellinger EP (2007). Necrotizing soft-tissue infection: diagnosis and management. Clin Infect Dis.

[10] Stevens DL, Bisno AL, Chambers HF (2014). Practice guidelines for the diagnosis and management of skin and soft tissue infections: 2014 update by the infectious diseases society of America. Clin Infect Dis.

[11] Wilkinson D, Doolette D (2004). Hyperbaric oxygen treatment and survival from necrotizing soft tissue infection. Arch Surg.

[12] Demello FJ, Haglin JJ, Hitchcock CR (1973). Comparative study of experimental Clostridium perfringens infection in dogs treated with antibiotics, surgery, and hyperbaric oxygen. Surgery.

